# Evaluation of Arterial Histopathology and microRNA Expression That Underlie Ultrasonography Findings in Temporal Arteries of Patients with Giant Cell Arteritis

**DOI:** 10.3390/ijms24021572

**Published:** 2023-01-13

**Authors:** Alen Suljič, Alojzija Hočevar, Vesna Jurčić, Luka Bolha

**Affiliations:** 1 Institute of Microbiology and Immunology, Faculty of Medicine, University of Ljubljana, 1000 Ljubljana, Slovenia; 2 Department of Rheumatology, University Medical Centre Ljubljana, 1000 Ljubljana, Slovenia; 3 Faculty of Medicine, University of Ljubljana, 1000 Ljubljana, Slovenia; 4 Institute of Pathology, Faculty of Medicine, University of Ljubljana, 1000 Ljubljana, Slovenia

**Keywords:** giant cell arteritis, ultrasonography, halo sign, arterial remodeling, temporal artery biopsy, microRNA, inflammation, immunopathology, diagnosis, vasculitis

## Abstract

The aim of this study was to assess the interrelation between vascular ultrasonography (US) findings, histopathological data, and the expression of selected dysregulated microRNAs (miRNAs) in giant cell arteritis (GCA). The study included data on the clinical parameters, US measurements, and temporal artery biopsies (TABs) of 46 treatment-naïve patients diagnosed with GCA and 22 age-matched non-GCA patient controls. We performed a comprehensive comparative and correlation analysis along with generation of receiver operating characteristic (ROC) curves to ascertain the diagnostic performance of US examination parameters and selected miRNAs for GCA diagnosis. We showed significant differences in the US-measured intima–media thickness of the temporal arteries, the presence of a halo sign, and the presence of luminal stenosis between GCA-positive/TAB-positive, GCA-positive/TAB-negative, and non-GCA patients. Correlation analysis revealed significant associations between several histopathological parameters, US-measured intima–media thickness, and the halo sign. We found that the significant overexpression of miR-146b-5p, miR-155-5p, miR-511-5p, and miR-21-5p, and the under-expression of the miR-143/145 cluster, miR-30a-5p, and miR-125a-5p, coincides and is associated with the presence of a halo sign in patients with GCA. Notably, we determined a high diagnostic performance of miR-146b-5p, miR-21-3p, and miR-21-5p expression profiles in discriminating GCA patients from non-GCA controls, suggesting their potential utilization as putative biomarkers of GCA. Taken together, our study provides an insight into the US-based diagnostic evaluation of GCA by revealing the complex interrelation of clearly defined image findings with underlying vascular immunopathology and altered arterial tissue-specific miRNA profiles.

## 1. Introduction

Giant cell arteritis (GCA) is the most common primary systemic vasculitis in adults older than 50 years [1,2]. It affects large and medium-sized arteries, particularly the extracranial branches of the carotid artery and the aorta. Ischemic complications resulting from the vasculitic occlusion of affected arteries represent a major disease burden, among which permanent vision loss is the most frequent [3]. The diagnosis of GCA is clinical; it is based on the presence of typical symptoms and signs along with elevated levels of inflammatory markers [4]. However, temporal artery biopsy (TAB) or appropriate imaging examination is required to make a confident diagnostic decision [5,6].

Ultrasonography (US) has become an increasingly valuable tool for the diagnosis of GCA. It has the advantage of being a non-invasive and relatively rapid procedure when performed with sufficient expertise [7]. The European Alliance of Associations for Rheumatology (EULAR) recommends the US examination of temporal and axillary arteries as the first imaging modality to be employed in suspected GCA cases [8]. The characteristic finding seen on US examination when assessing the affected temporal arteries (TAs) of GCA patients is the hypoechoic rim surrounding the vessel lumen, i.e., the “halo sign” [9,10], which represents the inflammatory thickening of the arterial wall [11]. Notably, US enables a precise quantitative assessment of the intima–media complex via the measurement of intima–media thickness [6,12]. In current clinical practice, US findings in patients with suspected GCA are conveyed as a binary result (positive or negative). Nevertheless, the variability of US measurements could reveal a relationship between the halo’s thickness and disease severity. For example, recent studies by van der Geest et al. demonstrated that the quantitative US halo score, measured from TAs, can serve as a marker for the presence and progression of GCA, namely, ischemic vision loss [13,14].

Until recently, a TAB served as the gold-standard test for the diagnosis of GCA [15]. Although the procedural risk of TAB is relatively low, its invasive nature carries an inherent risk and restricts resources [16]. The advantages of US examination over TAB include its wide availability, higher sensitivity, greater cost-effectiveness, and its facilitation of the ability to examine the entire length of an affected TA or other suspected arteries [17,18,19]. The advantages of imaging techniques over TAB are particularly evident in cases of suspected extracranial vasculitis of large vessels. In these cases, imaging modalities such as US, magnetic resonance imaging (MRI), or positron emission tomography with computed tomography (PET-CT) provide a reliable diagnostic option [20] and there are even efforts to characterize the vascular lesions associated with GCA via PET/MRI [21].

In general, US examination has been instrumental in defining GCA, and US halo scoring may allow for the quantification of the extent of vascular inflammation, intimal hyperplasia, and ischemic manifestations in GCA patients [13,14,22]. Nevertheless, despite substantial advances in disease management, morbidity remains high, while there is a lack of mechanistic understanding of GCA and relatively little insight into GCA-related vascular pathophysiology [19,22]. Therefore, an unprecedented need remains for the elucidation of GCA-specific pathogenic mechanisms and drivers of GCA-related vascular remodeling, which may differ between patients and lead to different clinical outcomes [10,15,23,24,25], thereby enabling earlier and more reliable GCA diagnosis and future treatment strategies with more personalized therapeutic approaches [19,22]. Considering the above-mentioned limitations with respect to GCA management, we hypothesized that a comprehensive, interconnected evaluation of patients’ clinical characteristics, TA-focused US imaging results, TAB-based histopathological assessments, and microRNA (miRNA)-based epigenetic aspects of GCA arterial lesions might provide a crucial insight into the US-based diagnostic evaluation of GCA. By a thorough US assessment of the TAs of treatment-naïve GCA patients, we related the obtained measurements to the quantitatively assessed histopathological features of TABs, which were obtained from these patients after US examination. Then, we investigated the relationship between the obtained US data and the altered miRNA profiles in the TABs from GCA patients, as altered miRNA expression has been strongly associated with the extent and severity of GCA-related arterial wall remodeling and inflammation in our previous studies [25,26]. Finally, we evaluated the diagnostic performance of US examination parameters and altered arterial miRNAs with respect to the diagnosis of GCA in our patient cohort, which confirmed the potential of the use of miRNAs as putative biomarkers in autoimmune diseases [27,28], including GCA [25,26,29,30].

## 2. Results

### 2.1. Patient Characteristics

Overall, we observed significant differences between the GCA and non-GCA patient groups in all the assessed histopathological and US parameters, except for the media thickness measurement based on the histopathological evaluation and the occurrence of extracranial involvement (Table 1). In addition, we observed strong significance regarding the presence of constitutional symptoms, erythrocyte sedimentation rate (ESR) and C-reactive protein (CRP) levels, and the white blood cell count. Conversely, we found no significant differences in age, sex, visual disturbances, the presence of polymyalgia rheumatica (PMR) symptoms, and hemoglobin levels between the non-GCA and GCA patients (Table 1). The detailed values of each diagnostic parameter and its respective significance are presented in Table 1.

### 2.2. Differences in US Examination Parameters between Patient Groups

**Figure 1:**
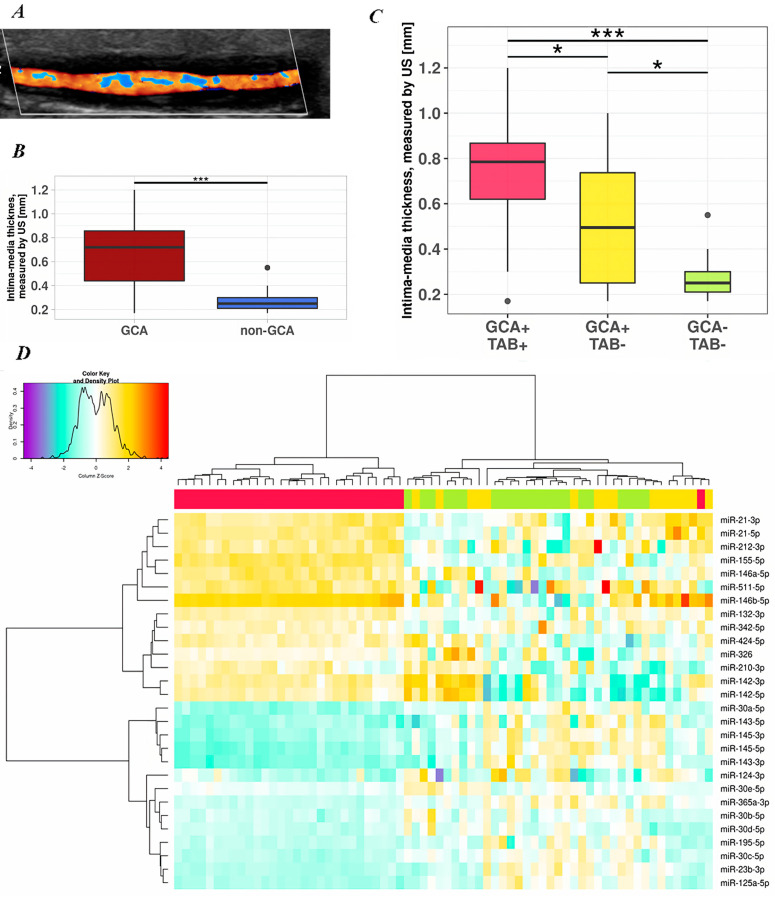
Longitudinal US cross section of TA in patient with GCA—a representative color Doppler US image, depicting the TA halo sign (courtesy of A. Hočevar, UMC Ljubljana) (**A**). Note the darker, hypoechoic bands adjacent to colored flow inside the lumen of the artery in the longitudinal plane. Intima–media thickness measured by US of each patient group (**B**,**C**). Horizontal line within the boxplots denotes the median and horizontal border lines represent the interquartile range. The numbers of patients in GCA+/TAB+, GCA+/TAB−, and non-GCA (GCA−/TAB−) patient groups were 30, 16, and 22, respectively. Statistical significance between patient groups was evaluated in a pairwise manner with Welch’s *t*-test (* *p* < 0.05; *** *p* < 0.001). Heatmap with unsupervised hierarchical clustering of 28 miRNAs with altered expression in TABs from GCA patients (**D**). Row Z-scores of log_2_ fold-change values are presented for each miRNA in GCA+/TAB+ (red cluster; n = 30) and GCA+/TAB− (yellow cluster; n = 16) patient subgroups and non-GCA patient controls (green cluster; n = 22). Patient subgroups were established based on the intima–media TA thickness measured by US.

Next, we found significant differences in the US-measured intima–media thickness between the GCA and non-GCA patients (*p* < 0.001). The median intima–media thickness measured by US was 0.72 mm (range 0.17–1.2 mm) in the GCA patients and 0.25 mm (range 0.17–0.55 mm) in the non-GCA patients (Figure 1B). Notably, the median intima–media thickness measured by US was 0.79 mm (range 0.17–1.2 mm) in the GCA+/TAB+ patients and 0.5 mm (range 0.17–1 mm) in the GCA+/TAB− patients. In addition, we found significant differences in the intima–media thickness between the GCA+/TAB− and non-GCA (GCA−/TAB−) patients (*p* = 0.02). The intima-media thickness measured by US in the patient groups and subgroups is depicted in Figure 1. The observed frequency of patients with a TA halo sign and luminal stenosis showed a significant difference between the GCA+/TAB+ and GCA+/TAB− patients (*p* < 0.01 and *p* = 0.02, respectively). The relationship between the frequency of the TA halo sign’s presence, extracranial involvement, and the occurrence of luminal stenosis in each patient subgroup is summarized in Table 2.

### 2.3. Differences in US-Measured Intima–Media Thickness between Patients with Different Clinical Characteristics and TAB-Derived Histopathological Features

Overall, a significant difference in the US-measured intima–media thickness was determined between the patients with absent/present clinically altered TAs (absent: median = 0.33 mm and range = 0.17–1 mm; present: median = 0.77 and range = 0.21–1.2 mm; *p* < 0.001). With regard to TAB histology, we found an association between intima–media thickness and the absence/presence of plasma cells (absent: median = 0.39 mm and range = 0.17–1.2; present: median = 0.81 mm and range = 0.3–1.14 mm; *p* < 0.001) and internal elastic lamina disruption (absent: median = 0.3 mm and range = 0.17–1 mm; present: median = 0.8 mm and range = 0.3–1.2 mm; *p* < 0.001). Conversely, no significant differences were found in the US-measured intima–media thickness between genders (male: median = 0.7 mm and range = 0.21–14 mm; female: median = 0.5 mm and range = 0.17–1.20 mm; *p* = 0.07), absence/presence of constitutional symptoms (absent: median = 0.45 mm and range = 0.17–0.64 mm; present: median = 0.68 mm and range = 0.17–1.2 mm; *p* = 0.08) and PMR symptoms (absent: median = 0.55 mm and range = 0.17–1.2 mm; present: median = 0.80 and range = 0.47–1 mm; *p* = 0.06).

### 2.4. miRNA Expression in TABs from GCA and Non-GCA Patients, Classified According to US-Measured Intima–Media TA Thickness

Differences between the miRNA expression levels in the TABs from the GCA and non-GCA patient subgroups, as determined by US-measured intima–media TA thickness, were assessed by unsupervised hierarchical clustering and are displayed as a heatmap in Figure 1D. Overall, analysis yielded two distinct clusters: one corresponding to the subgroup of GCA+/TAB+ patients and the other to the combined subgroup of GCA+/TAB− and non-GCA patients. Of note, one GCA+/TAB+ sample was clustered together with the GCA+/TAB− group (Figure 1D). Compared to the miRNA expression levels in the other patients comprising the GCA+/TAB+ subgroup, this sample had lower expression levels of miR-511-5p and miR-210-3p. Overall, the expression profiles of the included miRNAs showed a relatively high degree of heterogeneity in the GCA+/TAB− and non-GCA clusters compared to the GCA+/TAB+ cluster, and no clear patterns could be delineated between the GCA+/TAB− and non-GCA patients (Figure 1D).

### 2.5. Association between GCA Diagnosis and US Examination Parameters, Histological Parameters, and miRNA Expression Profiles

**Figure 2:**
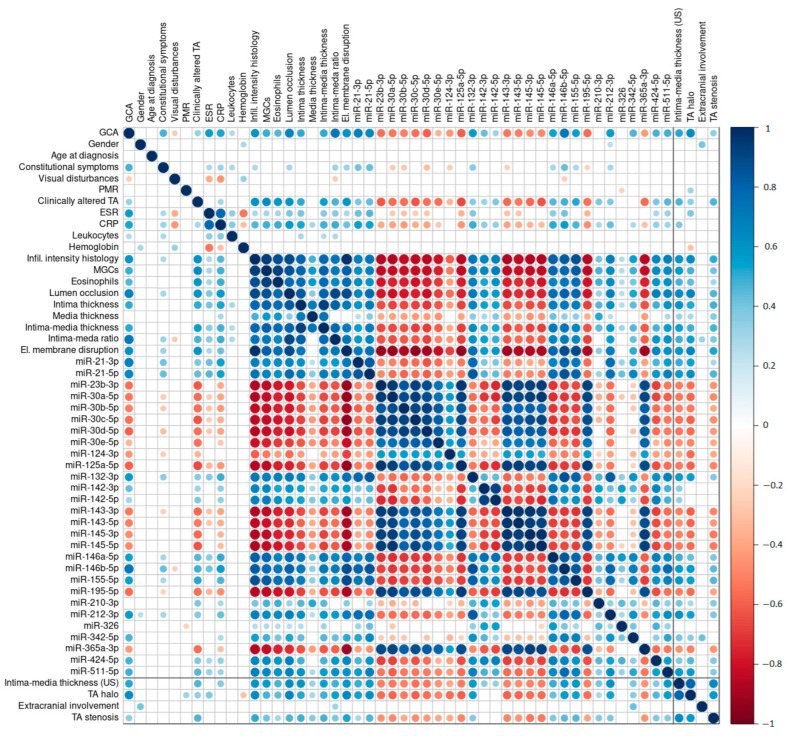
Correlation matrix of associations between all diagnostic parameters included in the study. Associations were calculated based on variable type combinations (nominal vs. nominal—φ; nominal vs. numeric—*r*; numeric vs. numeric—*ρ*). Blank squares represent non-significant associations. The direction and level of corresponding correlation coefficients are presented with colors (blue for positive correlation; red for negative correlation) and their respective intensities with circle size (size of the circle increases proportionally to the correlation intensity). Associations with US examination parameters are highlighted with black lines.

The final GCA diagnosis made by clinicians showed a strong significant, positive correlation with the histological assessment of the inflammatory infiltrate (*r* = 0.8), the intima–media thickness ratio (*r* = 0.72), and the expression levels of miR-146b-5p (*r* = 0.72), miR-21-3p (*r* = 0.68), and miR-21-5p (*r* = 0.64). The other studied parameters showed relatively modest correlations with the final GCA diagnosis (Figure 2). Demographic and clinical parameters showed weak correlations with the other assessed parameters (Figure 2), except clinically altered TAs (significant positive correlation with multinucleated giant cell (MGC) density, *r* = 0.62; significant negative correlation with expression level of miR-125a-5p, *r* = −0.66; miR-30c-5p and miR-145-5p, both *r* = −0.63).

**Figure 3:**
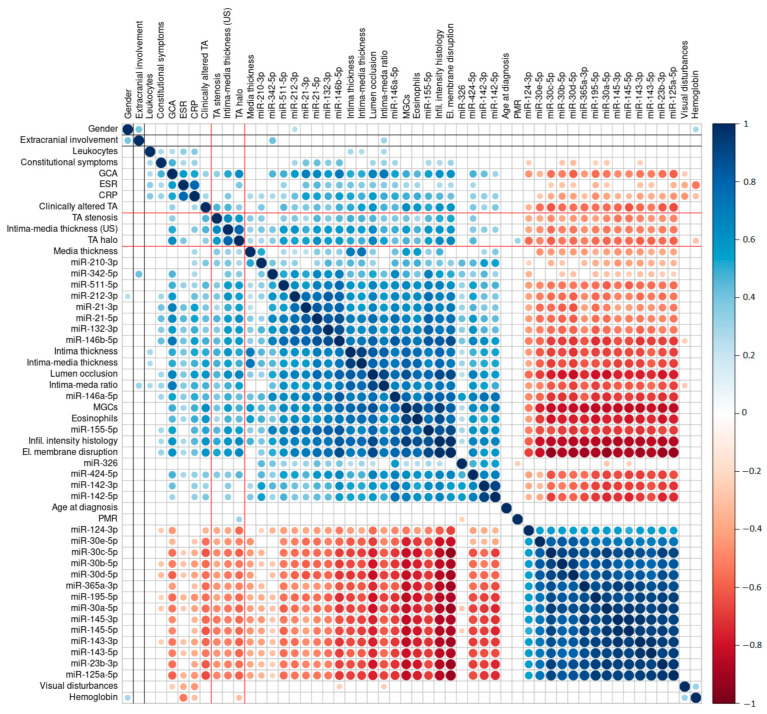
Correlation matrix of hierarchically clustered associations between all diagnostic parameters included in the study. Associations were calculated based on variable type combinations (nominal vs. nominal—φ; nominal vs. numeric—*r*; numeric vs. numeric—*ρ*). Blank squares represent non-significant associations. The direction and level of corresponding correlation coefficients are presented with colors (blue for positive correlation; red for negative correlation) and their respective intensities with circle size (size of the circle increases proportionally to the correlation intensity). Associations with US examination parameters are highlighted with black (extracranial involvement) and red (luminal stenosis, US-measured intima–media thickness, halo sign, and GCA diagnosis based on US examination) lines.

TAB histopathological parameters showed a strong intercorrelation pattern (Figure 3). The histological assessment of immune cell infiltrate intensity exhibited a strong positive correlation with the density of MGCs and the presence of a disruption of the internal elastic lamina (*ρ* = 0.93 and *r* = 0.97, respectively) and with the expression levels of miR-155-5p (*ρ* = 0.86). A significant negative correlation was observed between histologically assessed infiltrate intensity and pro-contractile vascular smooth muscle cell (VSMC) phenotype-related miRNAs [26] (mainly miR-23-3p and the miR-143/145 cluster; *ρ* = −0.87). A similar negative correlation (*ρ* = −0.87) was observed for miR-30a-5p. Similar to the TAB histopathological parameters, the parameters of the US examination also showed a strong intercorrelation.

Detailed data regarding the identified correlations between the parameters are presented in Figure 2 and Figure 3, and a comprehensive list of each paired comparison, with corresponding correlation coefficients and *p*-values, is provided in Table S1. The interrelation between US-measured intima–media thickness, TA halo sign presence, extracranial involvement, the presence of TA luminal stenosis, and other evaluated parameters is summarized below.

#### 2.5.1. US-Measured Intima–Media Thickness

Intima–media thickness measured by US showed a moderate significant, positive correlation with histologically assessed infiltrate intensity (*ρ* = 0.59), the histological measurement of intima–media thickness (*ρ* = 0.54), and internal elastic lamina disruption (*ρ* = 0.58). We observed a significant association of US-measured intima–media thickness with the expression profiles of all the included miRNAs except miR-326. The most notable were moderate positive correlations with the expression levels of miR-212-3p (*ρ* = 0.6), miR-155-5p (*ρ* = 0.58), and miR-511-5p (*ρ* = 0.58), and moderate negative correlations with the expression levels of four members of the miR-30 family (miR-30c-5p, *ρ* = −0.55; miR-30d-5p, *ρ* = −0.54; miR-30a-5p, *ρ* = −0.54; miR-30b-5p, *ρ* = −0.51) and miR-125a-5p (*ρ* = −0.53), miR-23b-3p (*ρ* = −0.52), miR-195-5p (*ρ* = −0.52), and the miR-143/145 cluster (*ρ* = −0.51). Detailed data regarding the determined correlations are presented in Figure 2 and Figure 3.

#### 2.5.2. The TA Halo Sign Presence

A moderate significant, positive correlation of the presence of a TA halo sign was observed with internal elastic lamina disruption (φ = 0.65), histopathologically assessed infiltrate intensity in TABs (*r* = 0.63), and final GCA diagnosis (φ = 0.62). We observed a significant association between the presence of the TA halo sign and the expression profiles of all the included miRNAs except miR-326. The most notable correlations of the TA halo sign with miRNA expression included moderate positive correlations with the expression levels of miR-21-5p (*r* = 0.61), miR-146b-5p (*r* = 0.57), and miR-132-3p (*r* = 0.55), and moderate negative correlations with the expression levels of miR-30c-5p, miR-125a-5p, and miR-143-5p (all *r* = −0.6). Detailed data regarding the determined correlations are presented in Figure 2 and Figure 3.

#### 2.5.3. Extracranial Involvement

We observed a significant positive correlation between extracranial involvement, the expression levels of miR-342-5p (*r* = 0.41), and gender (φ = 0.39). Overall, females displayed higher frequencies of extracranial involvement compared to males. Detailed data regarding the determined associations between extracranial involvement and other parameters are presented in Figure 2 and Figure 3.

#### 2.5.4. Presence of TA Luminal Stenosis

We observed poor correlation between TA luminal stenosis and other diagnostic parameters (Figure 2 and Figure 3). Interestingly, a comparison between US-assessed TA luminal stenosis and the luminal stenosis score based on TAB-based histopathological evaluation showed only a moderate positive correlation (*ρ* = 0.46).

### 2.6. Clinical Characteristics, TAB-Based Histopathological Features, and Differential miRNA Expression in Relation to Underlying US Examination Parameters

**Figure 4:**
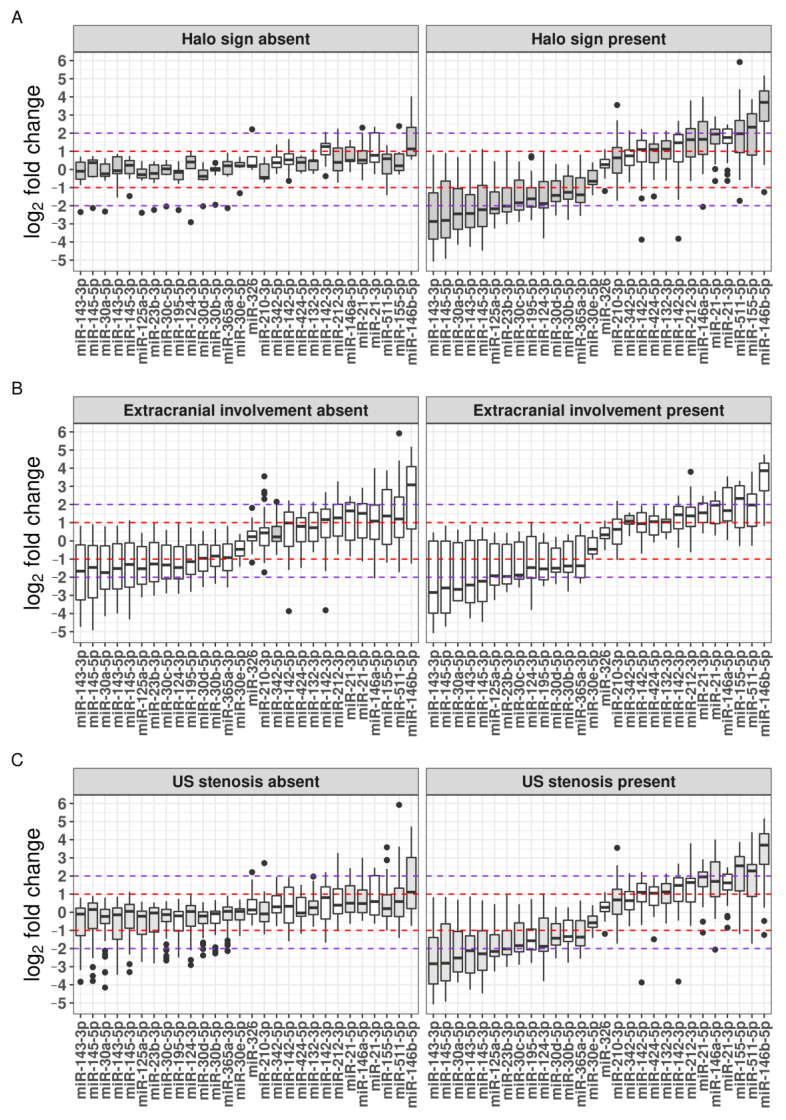
Expression profiles of 28 miRNAs in TABs from 46 GCA patients in relation to US examination parameters. Boxplots depict log_2_ fold-change values of each miRNA. Differential miRNA expression in patients with and without the TA halo sign present (**A**), in patients with and without extracranial involvement (**B**), and in patients with and without luminal stenosis (**C**). The red and violet dotted lines delineate two- and four-fold-change threshold of each miRNA, respectively. miRNAs exhibiting significant differential expression between patient groups are highlighted in grey.

We detected differential expression in relation to the presence of a TA halo sign of all the included miRNAs, except miR-142-5p and miR-326 (Figure 4A). The most notable significantly overexpressed (>4-fold) miRNAs in the patients with the TA halo sign were miR-146b-5p (median log_2_ fold-change (FC) = 3.7; *p* < 0.001), miR-155-5p (median log_2_ FC = 2.3; *p* < 0.001), miR-511-5p (median log_2_ FC = 2; *p* < 0.001), and miR-21-5p (median log_2_ FC = 2; *p* < 0.001) (Figure 4A). Conversely, the most notable significantly under-expressed (>4-fold) miRNAs included miR-143-3p (median log_2_ FC = −2.9; *p* < 0.001), miR-145-5p (median log_2_ FC = −2.8; *p* < 0.001), miR-30a-5p (median log_2_ FC = −2.5; *p* < 0.001), miR-143-5p (median log_2_ FC = −2.4; *p* < 0.001), miR-145-3p (median log_2_ FC = −2.2; *p* < 0.001), and miR-125a-5p (median log_2_ FC = −2.2; *p* < 0.001) (Figure 4A). Additionally, we observed a significantly higher occurrence of clinically altered TAs and significant differences in all the TAB-derived histopathological parameters, except the intima–media thickness ratio, in GCA patients with a TA halo sign (Table 3).

We determined no significant differences in the clinical and histopathological parameters between the GCA patients with and without extracranial involvement (Table 3). Overall, miR-342-5p was the only miRNA whose expression profiles differed between the patients with extracranial involvement and those without extracranial involvement (present: median log_2_ FC = 1.1; absent: median log_2_ FC = 0.23; *p* = 0.001) (Figure 4B).

We found significantly higher frequencies of stenosis seen in the US examination in the patients with clinically altered TAs (OR: 7.5, CI: 2–33, *p* = 0.001). Additionally, we detected significant differences in all the included TAB-based histopathological features in the patients with luminal stenosis observed by US compared to the patients with absent luminal stenosis, except with respect to eosinophil count and the intima–media thickness ratio (Table 3). Similarly, the expression profiles of all the included miRNAs differed in relation to the luminal stenosis, except for miR-342-5p, miR-326, and miR-21-3p (Figure 4C). Overall, patients characterized by the presence of the TA halo sign and luminal stenosis exhibited similar miRNA expression patterns (Figure 4A,C).

### 2.7. Discrepancies between GCA Diagnosis Based on TAB and TA US

We observed some disagreement between the diagnostic results obtained by TAB and US assessment. We noted nine GCA patients (two of them from the GCA+/TAB+ subgroup and seven from the GCA+/TAB− subgroup) that had negative US results for GCA. They were mostly female (8/9, 89%) with a reported presence of constitutional symptoms. Interestingly, one of the two patients from the GCA+/TAB+ subgroup was the patient that did not cluster with the rest of the subgroup, according to the miRNA expression profiles (Figure 1D). Conversely, we observed nine GCA patients from the GCA+/TAB− group, predominantly consisting of males (7/9, 78%), who had a TA halo sign present and five of them exhibited luminal stenosis (Table 2). Their mean US-measured TA intima–media thickness was 0.74 mm (range 0.47–1 mm). Notably, their TABs exhibited no characteristic signs of arterial wall inflammation.

### 2.8. Diagnostic Performance of US and Altered miRNAs in GCA Patients

The receiver operating characteristic (ROC) curve analysis showed a high discriminatory power of the TA halo sign (area under the ROC curve (AUC) = 0.90, 95% confidence interval (CI) = 0.87–0.99), intima–media thickness measured by US (AUC = 0.91, 95% CI = 0.78–0.99), and arterial tissue expression levels of miR-146b-5p (AUC = 0.95, 95% CI = 0.88–0.99), miR-21-3p (AUC = 0.91, 95% CI = 0.82–0.99), and miR-21-5p (AUC = 0.90, 95% CI = 0.80–0.99) for GCA classification. Notably, miR-146b-5p, miR-21-3p, and miR-21-5p exhibited the most prominent association with the final GCA diagnosis (*r* = 0.72, *r* = 0.68, and *r* = 0.64, respectively) (Figure 2). The highest accuracy was obtained with four predictors: TA halo sign (0.88), miR-21-3p expression levels (0.85), miR-146b-5p expression levels (0.84), and TA intima–media thickness measured by US (0.79). All four selected predictors showed high specificity (≥86%) and good sensitivity (≥75%) in discriminating GCA patients from non-GCA-afflicted controls. A comprehensive list of data on each predictor, including optimal cut-off values based on the Youden index, is presented in Table S2.

## 3. Discussion

In this study, we characterized the relationship between US findings and the underlying histological features of inflammatory vascular lesions in the TAs of GCA patients and explored different mediators that drive the immunopathology of and vascular remodeling in GCA. The main strength of our current study is its comprehensive association analysis between the US-based image assessment features of the TAs of treatment-naïve GCA patients and the features of TABs, which were obtained shortly after US examination. This enabled us to gain an optimal insight into the disease’s inflammation dynamics without the treatment-induced immunosuppressive effects. Furthermore, the US findings on TAs were used to explore the associations between the altered expression profiles of relevant miRNAs related to arterial wall remodeling and inflammation. This approach allowed us to gain insight into the GCA-related vascular pathophysiology that underpins the US diagnostic findings, crucially contributing to the accuracy of GCA diagnosis.

The comparison of the demographic, clinical, laboratory-based, and histopathological findings between the age-matched GCA patients and the control group confirmed known differences between GCA and non-GCA patients [4,10,18]. Along with differences in the presence of constitutional symptoms, visible TA swelling or tenderness, and the levels of acute phase reactants, we also determined significant differences in the TAB histopathological characteristics, comprising abundant numbers of lymphocytes and macrophages; the presence of MGCs, eosinophils, and myofibroblasts; the disruption of the internal elastic lamina; and the extent of intimal thickening, and these results were in accordance with the findings of previous studies [15,23,24]. The assessment of the clinical findings and TAB histopathology, as reference tests for GCA diagnosis, was extended with the inclusion of data obtained by US examination. Characteristic US TA halo signs and the TA intima–media thickness showed a significant differentiation between the TAB-positive GCA patients and the TAB-negative GCA patients. Overall, our results regarding the US-measured TA vessel-wall thicknesses aligned well with the results of a large multicentric study entitled “The Role of Ultrasound Compared to Biopsy of Temporal Arteries in the Diagnosis and Treatment of Giant Cell Arteritis” (TABUL) [17], a recent meta-analysis [31], and a diagnostic performance utility study on the thickness of the intima–media complex [32]. In these studies, a cut-off value of intima–media thickness was determined at 0.4 mm for GCA diagnosis based on US measurement. The studies suggest that a normal thickness of the intima–media complex in the TAs of adults aged ≥ 70 years measures approximately 0.2 mm through US, whereas the vessel-wall thickness of inflamed TAs commonly measures 0.5–0.9 mm [9,11,33]. Notably, we observed only a moderate association between US-measured intima–media thickness and intima–media thickness determined by TAB-based histopathological assessment, with a stronger association with intimal thickness (*ρ* = 0.59) compared to medial thickness (*ρ* = 0.32). Overall, our data suggest that TA vessel-wall thickening, which occurs due to enhanced inflammation, results predominantly in a greater expansion of the intima.

The utility of US for the examination of arteries along their length minimizes the risk of false-negative results, which constitute a crucial limitation of TAB [6,17,33], mostly due to skip lesions (foci of discontinuous inflammation) that occur in approximately 10% of cases [34]. In the TABUL study, the authors compared the diagnostic capability of TAB and US in patients with suspected GCA [17]. They described the lower sensitivity of a TAB (39%) in comparison to US (54%) and the higher specificity of a TAB (100%) compared to US (81%). In a recent meta-analysis [31], the authors reported a pooled sensitivity of 67% and specificity of 95% when using the TA halo sign as a diagnostic criterion for GCA. In another large meta-analysis performed by Rubenstein et al. [5], the authors estimated a TAB sensitivity of 77%. A study by Dua et al. [35] reported similar sensitivity and specificity of a TAB (61% and 98%, respectively) in patients wherein GCA diagnosis was based on clinical findings. Although the studies performed by Rubenstein et al. and Dua et al. [5,35] included a large total number of patients, they exhibited a high degree of heterogeneity between the included subjects, which could be partially related to a prevalent lack of details regarding the sampling and handling of TABs along with inconsistent histopathological measurements of inflammation parameters. Interestingly, similar heterogeneities were observed in the US examination of TAs, wherein US examination could not always detect arterial inflammation in TAB-positive GCA patients [5,6,31]. In our study, we observed high specificity and sensitivity of the TA halo sign (99% and 86%, respectively) as well as high specificity and sensitivity of TAB histopathological assessment (99% and 71%, respectively). Although our patient cohorts lacked the sheer numbers of included patients that meta-analyses provide, our results seem to adequately agree with their findings. Overall, the variability in the reported diagnostic capability of TAB and US examination implies that we cannot yet replace the more invasive methods, such as TAB, with non-invasive imaging modalities such as US. Furthermore, the findings of the mentioned studies support the idea that the TAB-negative GCA patient subgroup constitutes a separate phenotype, which requires further characterization [3].

To determine relations between the demographic, clinical, laboratory-based, histopathological, and US examination data and altered miRNA expression, we performed a comprehensive correlation analysis and revealed significant associations between the majority of the studied parameters (Figure 2 and Figure 3). Using hierarchical clustering, we identified three major clusters (Figure 3). The first cluster comprised strongly positively intercorrelated miRNAs, including miR-30 family members (miR-30a-5p/, -30b-5p/, -30c-5p/, -30d-5p/, and -30e-5p), miR-365a-3p, miR-195-5p, the miR-143/145 cluster (miR-143 and miR-145), miR-124-3p, miR-125a-3p, and miR-23b-3p. All of these miRNAs were under-expressed in GCA arterial lesions compared to the non-GCA controls and have been associated with the regulation of arterial wall remodeling, VSMC phenotypic modulation, and arterial inflammation [25,26,36]. The second cluster contained parameters that strongly negatively correlated with the first cluster. Among the most prominent were those previously quantified: the disruption of the internal elastic lamina; the overall infiltration intensity of immune cells; the presence of MGCs, plasma cells, and eosinophils; elevated numbers of nuclear factor of activated T cells; cytoplasmic 1 (NFATC)-positive (NFATC^+^) cells; CD3^+^, CD4^+^ and CD8^+^ T lymphocytes; CD20^+^ B lymphocytes; CD68^+^ macrophages; intimal thickening, and luminal stenosis [25]. Of the miRNAs, the second cluster contained miR-424-5p, miR-142-3p/-5p, miR-155-5p, miR-146a-5p, miR-146b-5p, miR-21-3p/-5p, miR-132-3p, miR-212-3p, miR-511-5p, miR-210-3p, miR-342-5p, and miR-326. The third cluster revealed a network of strongly positively interconnected parameters. The TA halo sign and US-measured intima–media thickness exhibited a positive correlation with acute phase reactants, histopathological parameters, and immune-related miRNAs described in the second cluster. We observed poor interrelation between extracranial involvement and the majority of the examined parameters, apart from a weak correlation with miR-342-5p expression levels, the intima–media thickness ratio, and male gender. Overall, these results suggest that US measurements relatively accurately reflect the intensity of T-cell immune response in affected TAs, as confirmed by TAB-based histopathological assessment. Moreover, the same US measurements corresponded well to altered miRNAs, which have been identified as key regulators of inflammation and arterial wall remodeling.

Notably, we determined significant differences in miRNA expression between the GCA patients in relation to the presence of the TA halo sign and TA luminal stenosis. In patients with the TA halo sign present, we determined an at least two-fold under-expression of pro-contractile VSMC phenotype-related miRNAs and an at least two-fold overexpression of immune-related miRNAs, as defined by previous studies [25,26]. Similar differences in miRNA expression were also observed in relation to the presence of TA luminal stenosis (Figure 4). Of the differentially expressed miRNAs, ten miRNAs exceeded the four-fold miRNA fold-change cut-off, including significantly overexpressed miR-146b-5p, miR-155-5p, miR-511-5p, and miR-21-5p, and significantly under-expressed miR-143-3p, miR-143-5p, miR-145-3p, miR-145-5p, miR-30a-5p, and miR-125a-5p (Figure 4). The dysregulation of these ten miRNAs has been associated with processes closely related to GCA vascular pathophysiology, predominantly with the modulation of the VSMC phenotype. Studies have shown that the upregulation of the miR-143/145 cluster is crucial for the maintenance of the physiologically normal “contractile” VSMC phenotype, whereas the downregulation of miR-143 and miR-145, which is mediated by vascular stress and corresponding elevated levels of platelet-derived growth factor (PDGF), resulted in the VSMC phenotype switching into the “synthetic” phenotype and podosome formation, initiating VSMC migration and neointima formation [37,38,39,40]. A similar impact on VSMC phenotype and intimal hyperplasia has been determined for miR-125a-5p [41] and several members of the miR-30 family [42], whose upregulation inhibited VSMC proliferation and migration and was associated with the inhibition of neointima formation. Conversely, the upregulation of miR-21 promoted VSMC proliferation and migration and was positively associated with neointima formation through the inhibition of PTEN, Bcl-2, and SPRY2 [43,44]. Moreover, the PDGF-induced upregulation of miR-146b-5p enhanced the proliferation and migration of stimulated VSMCs [45]. In addition, the overexpression of miR-155-5p and miR-511 has been linked to enhanced T-cell activation and proliferation and macrophage inflammatory responses in GCA-affected temporal arteries [25].

In GCA lesions, the transmural inflammation of affected arterial walls ultimately results in the formation of granulomas with MGCs, wherein abundant numbers of IFN-γ-activated macrophages stimulate the induction and production of pro-inflammatory cytokines, chemokines, reactive oxygen species, matrix metalloproteinases, and growth factors, of which the later essentially comprise vascular endothelial growth factor (VEGF), PDGF, and transforming growth factor beta (TGF-β). These events lead to the degradation of the internal elastic lamina and mediate VSMC phenotypic modulation from a contractile/differentiated phenotype into a synthetic/proliferative highly migratory phenotype. These “synthetic” VSMCs then migrate from the media towards the vessel lumen, resulting in intimal hyperplasia and vessel occlusion [19,22,46,47,48]. Since the downregulation of pro-contractile and the upregulation of pro-synthetic VSMC phenotype-related miRNAs has been linked to proliferative neointima formation [26,49,50], our results suggest that the expression patterns of distinct miRNAs may influence the TA halo sign and luminal stenosis in GCA and enable effective differentiation between GCA and non-GCA patients. Thus, the utility of the US TA halo score in identifying GCA patients with intimal hyperplasia and ischemic manifestations [14] can be additionally enhanced by employing epigenetics (e.g., miRNA dysregulation) in the US assessment of patients with suspected GCA, along with TAB histopathological evaluation. According to unsupervised hierarchical clustering, a specific miRNA expression pattern could discern our GCA+/TAB+ subgroup from other included patients (Figure 1D). Overall, a highly heterogeneous expression pattern of these miRNAs in the GCA+/TAB− and non-GCA subgroups could indicate a more dynamic phase of arterial wall remodeling in the earlier stages of the disease, and we speculate that a distinctive pattern could have been discerned if we employed a larger sample size.

To evaluate the diagnostic performance of the included miRNAs and US examination parameters in diagnosing GCA, we performed an ROC curve analysis for the US-measured TA intima–media thickness, TA halo sign, TA luminal stenosis, and expression profiles of all 28 included miRNAs (Table S2). Overall, the comparison between the US-derived measurements and the miRNA expression profiles showed a comparable discriminatory power between parameters, with AUC estimates ranging from 0.67 to 0.95 (Table S2). The determined cut-off value of 0.56 mm for the US-measured TA intima–media thickness was slightly higher compared to the two most recent studies, which reported cut-offs at 0.4 mm and 0.42 mm, respectively [32,51]. This discrepancy was likely due to the difference in the number of included patients and due to the classifying algorithm used for the ROC curve analysis (Random Forest classifier was shown to outperform Logistic Regression in terms of accuracy [52]). The diagnostic performance of the TA halo sign as a standalone criterion for the diagnosis of cranial GCA agreed well with recent findings concerning the role of the TA halo sign in GCA diagnosis [13,14,32]. Notably, the expression levels of miR-146b-5p, miR-21-3p, and miR-21-5p exhibited high diagnostic performance, with AUC values of 0.95, 0.91, and 0.91, respectively. Thus, our results indicate that the expression levels of certain altered arterial miRNAs could serve as potential diagnostic markers on a comparable level to that of the TA halo sign [4,13]. Overall, miR-146b-5p slightly outperformed miR-21-3p in terms of sensitivity, which could be observed from the lower cut-off value based on the Youden index. Regarding the diagnostic performance of the demographic, clinical, and laboratory data, no individual parameter could unambiguously confirm or reject GCA diagnosis in our study, which corresponds to the results obtained in previous studies [4]. Although miR-21 is among the crucial miRNAs that regulate pro-inflammatory responses, the VSMC phenotype, and intimal hyperplasia [43,44,53,54], its expression lacks specificity and thus limits its usefulness as a biomarker [55]. Nevertheless, the potential for detecting dysregulated circulating miRNAs in plasma and/or serum designates them as prime contenders for further research as diagnostic and prognostic biomarkers of GCA.

Although our study includes a comprehensive analysis of multiple diagnostic parameters from the TABs of treatment-naïve patients, its relatively small number of studied patients represents a major drawback for further in-depth analysis. This limitation also obstructs the effort to generalize our findings to different geographical areas or/and cohorts of GCA patients. Moreover, the inclusion of data on cytokine dynamics and miRNA levels, and their expression profiles in blood cells, plasma, and serum, which, crucially, would enable lower invasiveness of the GCA diagnostic procedure, would significantly improve our current single-center study. Lastly, the inclusion of imaging findings from different imaging modalities, such as MRI/magnetic resonance angiography (MRA) or PET-CT, in diagnostic performance analyses would expand the diagnostic horizon to other modalities in imaging repertoire. Nevertheless, our approach effectively sheds light on an intricate network of overlapping diagnostic parameters in GCA.

## 4. Materials and Methods

### 4.1. Patients

This retrospective study included 46 treatment-naïve patients diagnosed with GCA between September 2011 and December 2015 according to the American College of Rheumatology 1990 classification criteria [24,55]. The control non-GCA cohort included 22 treatment-naïve, age-matched patients with clinical suspicion of GCA, which was refuted after a complete patient work-up and follow-up. All patients were subjected to the fast-track GCA pathway, which consisted of evaluation at the Department of Rheumatology, University Medical Centre (UMC) Ljubljana, within 24 h of referral and immediate color Doppler US scan prior to TAB. Therapy was initiated after GCA diagnosis on the same day. TABs from enrolled patients were included in the analysis, comprising 30 histologically positive and 16 negative TABs from patients with GCA, and 22 negative TABs from non-GCA patient controls.

### 4.2. Clinical Parameters and US Examination Procedure

A detailed baseline evaluation protocol for GCA was routinely followed. It consisted of a structured medical history, clinical examination, and laboratory work-up, including determination of ESR, CRP levels, and total blood count. Baseline US examination included imaging of bilateral temporal and axillary artery territories, followed by one-side TAB.

A single, experienced ultrasonographer performed the US examination of temporal and axillary arteries prior to TAB, using Philips ATL HDI 5000 Ultrasound Machine (Philips, Amsterdam, Netherlands) with 5–17.5 and 5–12 MHz multi-frequency linear probes. The adjustable settings of the US machine were constant across all examinations. Color Doppler frequency was 7.0–9.3 MHz (pulse repetition frequency was 2.2–2.3 kHz) and 5.0–7.0 MHz (pulse repetition frequency was 3.5 kHz) for temporal and axillary arteries, respectively. Arteries were assessed in two planes (longitudinal and transverse) for the halo sign, along with intima–media measurement and assessment of luminal stenosis [8,11,32].

### 4.3. TAB Histopathological Assessment and miRNA Expression Analysis

In order to associate US measurements with arterial wall histopathological features and arterial tissue-specific miRNA alteration in TABs from enrolled GCA patients, we included the following parameters:

(a) Nine quantitatively assessed histopathological parameters, including inflammatory infiltrate intensity; extent of luminal stenosis; intima, media, and intima–media thickness; ratio between the intima and media thickness; internal elastic lamina disruption; and densities of MGCs and eosinophils per mm^2^.
(b) Expression levels of 28 miRNAs, specifically, miR-21-3p, miR-21-5p, miR-23b-3p, miR-30a-5p, miR-30b-5p, miR-30c-5p, miR-30d-5p, miR-30e-5p, miR-124-3p, miR-125a-5p, miR-132-3p, miR-142-3p, miR-142-5p, miR-143-3p, miR-143-5p, miR-145-3p, miR-145-5p, miR-146a-5p, miR-146b-5p, miR-155-5p, miR-195-5p, miR-210-3p, miR-212-3p, miR-326, miR-342-5p, miR-365a-3p, miR-424-5p, and miR-511-5p.

Data on quantitatively assessed histopathological features and altered miRNA expression levels in TABs from treatment-naïve GCA and non-GCA patients were obtained from our previously published work [25,26].

### 4.4. Statistical Analysis

Statistical analyses were performed in R software (version 4.2.1, R Foundation for Statistical Computing, Vienna, Austria) [56]. To assess the normality of data distribution, we used Q–Q plots and the Shapiro–Wilk test. The distribution of variables influenced the decision regarding the choice of parametric or non-parametric statistical tests.

To test the differences between the GCA and non-GCA groups, we used the Wilcoxon rank sum (Mann–Whitney) test for numeric (age at diagnosis, ESR, CRP, leukocyte and hemoglobin levels, overall histological assessment score of infiltrate intensity, MGCs, eosinophils and luminal stenosis scores, intima thickness, media thickness, intima–media thickness measurements and intima–media thickness ratio, and expression levels of 28 miRNAs and US-measured intima–media thickness) and Fisher’s exact test for the dichotomous categorical variables (GCA diagnosis, gender, presence of constitutional and PMR symptoms, clinically altered TAs, internal elastic lamina disruption, TA halo sign presence, extracranial involvement, luminal stenosis, and positive GCA diagnosis based on US examination).

To further explore the association between TAB-based histological and TA US-derived features, we divided the GCA group into histologically positive (GCA+/TAB+) and histologically negative (GCA+/TAB−) subgroups. We evaluated differences in US-measured intima–media thickness between GCA+/TAB+, GCA+/TAB−, and histologically negative non-GCA (GCA−/TAB−) patient subgroups with Welch’s *t*-test in a pairwise manner. To evaluate differences in the presence of the TA halo sign, extracranial involvement, luminal stenosis present on US, and positive GCA diagnosis based on US examination between GCA+/TAB+, GCA+/TAB−, and GCA−/TAB− patient subgroups, we used Fisher’s exact test. For the post hoc evaluation of differences between individual patient groups, we used the pairwise Fisher’s test. In multiple comparison analyses, we used the false discovery rate (FDR) as the method for multiple comparison correction.

Heatmap with unsupervised hierarchical clustering, employing log_2_ fold-change values of each miRNA per sample, was generated using RStudio v2022.2.0.443 software with the heatmap.2 function from the gplots v3.1.3 package [57].

Associations between numeric variables were evaluated with Spearman’s (*ρ*) rank correlation coefficient. Associations between numeric and categorical variables were assessed with point–biserial correlation using Pearson’s correlation coefficient (*r*). To assess the associations between categorical variables, we used the phi correlation coefficient (φ) and Fisher’s exact test. Hierarchical clustering of correlation coefficients was performed with Ward’s minimum variance method.

To determine the diagnostic performance of variables with respect to GCA diagnosis, we employed the ROC curve analysis. Prior to modelling, we preprocessed the input data (centering and scaling the variables to mean 0 and standard deviation to 1) to improve numeric stability. To study the diagnostic performance of US-measured intima–media thickness, TA halo sign, and expression profiles of selected miRNAs, we opted for random forest classifier. The dataset was randomly partitioned into four equally sized subsamples. One subsample was retained as testing data, and the other three were used to train the model. To account for differences in random subsampling, a 10-fold cross-validation was used, which was repeated 10 times. For comparison of diagnostic capabilities, we calculated the AUC and its 95% CI for each chosen diagnostic parameter using the stratified bootstrap method. Additionally, we determined the models’ accuracy, sensitivity, and specificity, along with the optimal cut-off value according to the Youden metric (J-index).

The threshold for statistical significance was set at *p* < 0.05 in all cases.

## 5. Conclusions

In summary, this study provides insight into the US diagnostic evaluation of TAs in GCA by revealing a complex interrelation of clearly defined image findings with underlying vascular immunopathology, which is described with histopathological methods. Furthermore, we showed that the altered expression of arterial tissue-specific GCA-related miRNAs is highly associated with US-based measurements. Overall, the combination of imaging, histopathological, and epigenetic data may eventually lead to further in-depth studies, which are crucial for the elucidation of the cause of the differences in the inflammatory infiltrate composition between GCA patients (which is characterized by different clinical outcomes), and aid GCA’s diagnosis, prognosis, treatment, and management.

## Figures and Tables

**Table 1:** Patient characteristics for each set of diagnostic parameters.

	Non-GCA Patients [n = 22] ^a^	GCA Patients [n = 46] ^b^
**Demographics**		
Age at diagnosis (years); median (range)	73 (58–87)	73 (55–92)
Gender (female); n (%)	18 (82)	30 (65)
**Symptoms and signs**		
Constitutional symptoms; n (%)	5 (23)	34 (74) ***
Visual disturbances; n (%)	11 (52)	12 (26)
PMR; n (%)	3 (18)	9 (20)
Clinically altered TA; n (%)	6 (32)	29 (63) *
**Laboratory**		
ESR (mm/h); median (range)	35 (11–113)	81 (28–130) ***
CRP (mg/ml); median (range)	5 (0–83)	68 (7–218) ***
Leukocytes (10^8^ cells); median (range)	7.4 (5–16)	9.5 (6–18) *
Hemoglobin (g/dl); median (range)	122 (101–156)	120 (99–140)
**Histopathology**		
Infiltrate intensity (score); median (range)	0 (0–0)	3 (0–4) ***
MGCs (score); median (range)	0 (0–0)	1 (0–4) ***
Eosinophils (score); median (range)	0 (0–0)	1 (0–3) ***
Luminal stenosis (score); median (range)	1 (1–1)	3 (1–4) ***
Intima thickness (mm); median (range)	0.1 (0.05–0.6)	0.35 (0.06–1.2) ***
Media thickness (mm); median (range)	0.12 (0.05–0.8)	0.15 (0.05–0.35)
Intima–media thickness (mm); median (range)	0.22 (0.1–0.6)	0.48 (0.11–1.55) ***
Intima–media ratio; median (range)	0.85 (0.5–1.5)	2.5 (0.7–4) ***
El. Membrane disruption; n (%)	0	29 (63) ***
**Ultrasonography**		
TA halo; n (%)	0	37 (80) ***
Extracranial involvement; n (%)	0	15 (36)
TA stenosis n; (%)	2 (17)	27 (59) *

GCA, giant cell arteritis; PMR, polymyalgia rheumatica; TA, temporal artery; ESR, erythrocyte sedimentation rate; CRP, C-reactive protein; MGC, multinucleated giant cell. Data were evaluated with Wilcoxon rank sum test or Fisher’s exact test. Statistical significance is conveyed by an asterisk (* *p* < 0.05; *** *p* < 0.001). ^a^ Missing data for constitutional symptoms, PMR, and clinically altered TA (n = 1); TA halo (n = 13); extracranial involvement (n = 14); and TA stenosis (n = 8). ^b^ Missing data for extracranial involvement (n = 4).

**Table 2:** Frequencies for each US measurement in each patient subgroup.

US Examination Parameter	GCA+/ TAB+ (n = 30)	GCA+/ TAB− (n = 16) ^a^	GCA−/ TAB− (n = 22) ^b^
TA halo sign; n (%)	28 (93)	9 (56) **	0
TA stenosis; n (%)	22 (73)	5 (31) *	2 (17)
Extracranial involvement; n (%)	11 (37)	4 (33)	0

GCA, giant cell arteritis; TAB, temporal artery biopsy; TA, temporal artery; US ultrasonography. Data were evaluated with Wilcoxon rank sum test or Fisher’s exact test. Statistical significance is conveyed by an asterisk (* *p* < 0.05, ** *p* < 0.01). ^a^ Missing data for extracranial involvement (n = 4). ^b^ Missing data for the TA halo (n = 12), TA stenosis (n = 8), and extracranial involvement (n = 13).

**Table 3:** TAB histopathology in relation to US examination parameters in GCA patients.

	Presence of the TA Halo Sign (n = 37)	Extracranial Involvement (n = 15) ^a^	Presence of US Stenosis (n = 27)
**Demographics**			
Gender (female); n (%)	22 (60)	14 (93) **	17 (63)
**Symptoms and signs**			
PMR; n (%)	9 (24)	3 (20)	6 (22)
Clinically altered TA; n (%)	28 (76) ***	8 (53)	22 (81) ***
**Laboratory**			
ESR (mm/h); median (range)	83 (28–130)	89 (28–120)	78 (28–120)
CRP (mg/ml); median (range)	68 (10–218)	76 (12–212)	66 (12–212)
Hemoglobin (g/dl); median (range)	119 (99–140)	116 (99–135)	120 (99–140)
**Histopathology**			
Infiltrate intensity (score); median (range)	3 (0–4) **	4 (0–4)	4 (0–4) **
MGCs (score); median (range)	2 (0–4) ***	4 (0–4)	2 (0–4) ***
Eosinophils (score); median (range)	1 (0–3) *	1 (0–3)	1 (0–3)
El. membrane disruption; n (%)	28 (76) ***	10 (75)	2 (81) **
Luminal stenosis (score); median (range)	4 (1–4) ***	4 (1–4)	4 (1–4) *
Intima thickness (mm); median (range)	0.4 (0.15–1.2) **	0.4 (0.09–0.8)	0.41 (0.17–1.2) **
Media thickness (mm); median (range)	0.15 (0.0 –0.35) **	0.16 (0.06–0.25)	0.2 (0.06–0.35) *
Intima–media thickness (mm); median (range)	0.55 (0.2–1.55) **	0.55 (0.22–1.05)	0.6 (0.23–1.55) **
Intima–media ratio; median (range)	2.5 (0.9–4)	3.0 (0.7–4)	2.5 (1.6–4)

GCA, giant cell arteritis; TAB, temporal artery biopsy; US, ultrasonography; TA, temporal artery; PMR, polymyalgia rheumatica; ESR, erythrocyte sedimentation rate; CRP, C-reactive protein; MGC, multinucleated giant cell. The differences between demographic, clinical, and TAB-based histopathological diagnostic parameters and US examination parameters of 46 GCA patients were evaluated with Wilcoxon rank sum test or Fisher’s exact test. Statistical significance is conveyed by an asterisk (* *p* < 0.05; ** *p* < 0.01; *** *p* < 0.001). ^a^ Missing data (n = 4).

## Data Availability

All data relevant to the study are included in the article or uploaded as Supplementary Material.

## Supplementary Materials

The following supporting information can be downloaded at: https://www.mdpi.com/article/10.3390/ijms24021572/s1.
